# A Tale of Immune-Related Adverse Events With Sequential Trials of Checkpoint Inhibitors in a Patient With Metastatic Renal Cell Carcinoma

**DOI:** 10.7759/cureus.8395

**Published:** 2020-06-01

**Authors:** Vinit Singh, Yvonne Chu, Varsha Gupta, Charlie Weige Zhao

**Affiliations:** 1 Internal Medicine, Monmouth Medical Center, Long Branch, USA; 2 Medical Oncology, Yale University School of Medicine, New Haven, USA; 3 Hospital Medicine, Yale New Haven Hospital, New Haven, USA; 4 Internal Medicine, Jersey Shore University Medical Center, Neptune City, USA; 5 Internal Medicine, Yale University School of Medicine, New Haven, USA

**Keywords:** immune-related adverse events, type i diabetes mellitus, immune checkpoint inhibitors

## Abstract

Immune checkpoint inhibitor (CPI) therapy is approved for the treatment of many cancers. As its use becomes more prevalent, sequential trials with different CPIs as monotherapy or combination therapy will become more common. It is thought that the increased cumulative dose of CPIs over multiple trials increases the risk of immune-related adverse events (irAEs). However, it is not known if using one CPI combination increases the risk of developing irAEs during the subsequent trial of a different CPI combination. Here, we present a patient with multiple episodes of high-grade irAEs over the course of sequential trials of combination CPIs. A 65-year-old female patient with metastatic renal cell cancer received two trials of combination CPIs. During the first trial with durvalumab and tremelimumab, she had CPI-induced grade 2 skin rashes and primary hypothyroidism with a mild elevation in lipase, normal antithyroid antibody profile, and normal blood glucose. Due to progression after the first trial, her regimen was changed to ipilimumab and nivolumab combination therapy. She subsequently presented to the emergency room with diabetic ketoacidosis on the sixth week following treatment initiation and was diagnosed with new-onset insulin-dependent type 1 diabetes mellitus (DM) with a negative antibody profile for DM. Immune CPIs cause irAEs by increasing immune activity against self-antigens. Sequential trials of CPIs may increase the risk of irAEs by increasing the cumulative CPI dose, or by organ injury inflicted by the first set of CPIs which is tipped “over the edge” by subsequent trials. We believe that the latter mechanism could be responsible for our case. Sequential CPI therapy should be planned carefully with increased surveillance for the early diagnosis and treatment of irAEs.

## Introduction

Immunotherapy is changing the landscape of cancer therapy. Among several immunotherapy options, checkpoint inhibitors (CPIs) are becoming a mainstay for cancer treatment. After the approval of ipilimumab for melanoma, several CPIs have been approved for cancers of the lung, kidney, renal, and colon, among others. As a result, the life expectancy of patients with cancer has improved significantly.

CPIs are monoclonal antibodies targeting CTLA-4, PD1, or PD-L1 receptors, restoring the anti-tumor activity of the immune system. The primary role of these receptors is to induce immune tolerance and protect the body from autoimmunity. CPI binding against these receptors increases T-cell-mediated anti-tumor activity [1].

A combination of multiple immunotherapy agents has been shown to have a higher immunogenic effect than single-agent therapy [2]. In fact, trialing different combinations of CPIs sequentially has been shown to be effective in some cancers [3]. However, this is currently not a recommended treatment approach due to the increased incidence of immune-related adverse events (irAEs). Of note, CPIs are not all equally active against all cancer types, nor are they equally likely to cause irAEs [4].

irAEs are graded into four categories where grades 3 and 4 are considered high-grade irAEs that may necessitate treatment cessation [5]. The incidence of irAEs varies from 15% to 90%, with 10%-20% being high-grade irAEs in monotherapy and 55%-60% in combination therapy [6]. This high incidence of high-grade irAEs questions the tolerability and usability of combination CPIs for cancer treatment. Research and trials are ongoing to gain a better understanding of the pathophysiology and management of this phenomenon. 

Here, we present a case of a patient who underwent sequential combination CPI immunotherapy with two different combinations for metastatic renal cell cancer and experienced grade 2 to 4 irAEs over the treatment duration. Through this case, we explore whether one CPI combination therapy can predispose patients to irAEs during the subsequent trial of CPIs.

## Case presentation

A 65-year-old female was diagnosed with metastatic oncocytic renal cell carcinoma (T4N1M1) with metastases to the liver, lung, and mediastinal lymph node. She was started on durvalumab and tremelimumab (four cycles) followed by durvalumab alone (12 cycles) for a total of 16 cycles over two years (Table 1). At the end of the first week of cycle 1, she developed grade 2 maculopapular rashes over her chest, back, abdomen, and legs, which was treated with pramoxine lotion and antihistamine syrup. Staging imaging done at the end of cycle 6 showed stable imaging findings. After cycle 8, she started feeling fatigued and had upper limb weakness. On routine monitoring, her thyroid-stimulating hormone (TSH) was found to be elevated to 11.29 µIU/ml with normal free T4 (0.92 ng/dl) and no detectable antithyroid antibodies. She was diagnosed with CPI-induced primary hypothyroidism. She was started on thyroid hormone replacement therapy and responded well. After cycle 14 with durvalumab alone, metastasis in her liver and mediastinal lymph node was found to have increased in size. Her treatment with durvalumab was aborted. As she was not eligible for any open clinical trials, she was started on ipilimumab and nivolumab combination therapy every third week after two months of the washout period. Twelve days after cycle 4 of ipilimumab and nivolumab combination therapy, she presented to the emergency room with nausea, vomiting, diarrhea, and abdominal cramps. She had influenza five days prior and was not taking any medication for it.

**Table 1:** Type, cumulative dose, duration, and sequence of checkpoint inhibitors from the beginning of cancer treatment and adverse events during the course of treatment.

Checkpoint inhibitor combinations	Adverse event	Time after initiating therapy
Durvalumab (1,500 mg x 14 cycles = 21,000 mg) and tremelimumab (75 mg x 4 cycles = 300 mg)	Grade 2 skin rashes	7 days
	Primary hypothyroidism	195 days
Ipilimumab (55 mg x 4 cycles = 220 mg) and nivolumab (160 mg x 4 mg = 640 mg)	Insulin-dependent type 1 diabetes mellitus	74 days

On physical examination, her vitals were normal. Laboratory investigation revealed that her glucose was elevated to 603 mg/dl (Table 2). Arterial blood gas showed a pH of 7.03, bicarbonate of 7 mmol/dl, and an anion gap of 25 mmol/dl. She was diagnosed with diabetic ketoacidosis (DKA). Besides the episode of CPI-induced primary hypothyroidism, her past medical history was otherwise significant for essential hypertension. At the time of presentation, her C-peptide level was decreased at 0.1 mg/dl and HbA1C was elevated to 8.5%.

**Table 2:** Blood biochemical and urinalysis results at the time of presentation in the emergency room.

	Results	Normal range
Blood biochemistry		
Sodium	122 mmol/L	135–145 mmol/L
Chloride	90 mmol/L	98–107 mmol/L
Potassium	4.6 mmol/L	3.3–5.0 mmol/L
Bicarbonate	7 mmol/L	21–32 mmol/L
Blood pH	7.03 units	7.32–7.43 units
Anion gap	25 mmol/L	5–15 mmol/L
Blood sugar	603 mmol/L	65–110 mg/dl
Urinalysis		
pH	5.5 units	5.5–7.5 units
Protein	1+	Negative-trace
Glucose	4+	Negative
Ketones	4+	Negative

**Figure 1:**
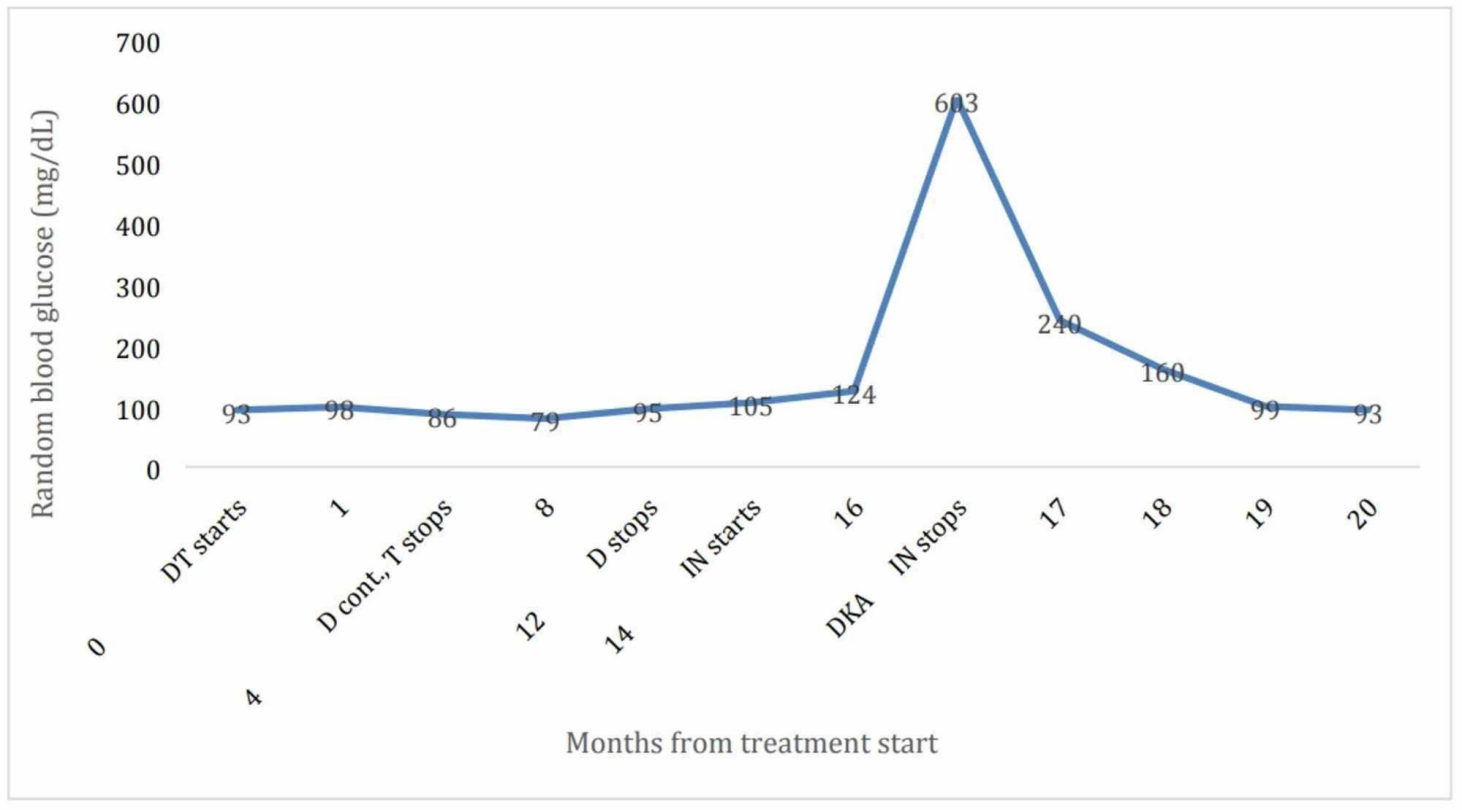
Random blood glucose of the patient over complete treatment cycles. Months are scaled for the treatment cycle. DT: durvalumab and tremelimumab combination, D: durvalumab alone, T: tremelimumab alone, IN: ipilimumab and nivolumab combination, DKA: diabetic ketoacidosis.

Her random blood glucose over the past eight months is shown in Figure 1. On further workup for autoimmune type 1 diabetes mellitus (DM1), testing for insulin autoantibodies, IA2, GAD68, and Zinc 8, was negative (Table 3). HLA testing was not performed. 

**Table 3:** Important biomarkers for assessment of new-onset type 1 diabetes mellitus in old age. IA-2 antibody: islet antigen 2 antibody, anti-GAD65 antibody: anti-glutamic acid decarboxylase antibody.

Biomarker	Results	Normal
C-peptide level	0.1 ng/ml	0.9–7.1 ng/ml
Glucagon level	17 pg/ml	8–57 pg/ml
Anti-GAD65 antibody	<5 IU/ml	<5 IU/ml
Insulin antibody	<0.4 U/ml	<0.4 U/ml
IA-2 antibody	<0.8 U/ml	<0.8 U/ml
Zinc transporter 8 antibody	<10 U/ml	<15 U/ml
Amylase	53 U/L	28–100 U/L
Lipase	62 U/L	<60 U/L

She was managed with aggressive IV fluids and the insulin drip. She was transitioned to subcutaneous insulin once her anion gap was closed. After two days, the patient was discharged on a basal-bolus insulin regimen after diabetes education prior to discharge from the hospital. No immunosuppression was given during the course of treatment. CPIs were discontinued after these episodes. After discharge, she underwent multiple sessions of embolization of liver metastasis and renal cancer. She was undergoing lenvatinib and everolimus trial for her renal cancer treatment. Fourteen months following her discharge from the hospital, she continues to be on a basal-bolus insulin regimen and her hemoglobin A1C was 5.1%.

## Discussion

We have described a patient with metastatic renal cell carcinoma who was diagnosed with grade 2-4 irAEs over the course of two sequentially administered combination CPIs. We propose the hypothesis that this presents a unique scenario where CPIs used during initial treatment predisposed the patient to develop a high-grade irAE during the subsequent trial.

Our patient first developed a grade 2 maculopapular rash within two weeks of CPI therapy initiation. This common side effect affects 60% of patients undergoing CPI immunotherapy and is used as a marker of increased immune activity, significantly related to progression-free survival in patients with melanoma and non-small cell lung cancer [7].

As treatment continued, the patient developed primary hypothyroidism which required thyroid replacement therapy. In previous clinical trials, thyroid-related disorders have been reported in 4% of patients taking tremelimumab [8]. For combination therapy, the incidence of hypothyroidism has a wide variability, ranging from 9% to 22% [9]. The mechanism is due to either central hypothyroidism caused by concurrent hypophysitis or direct injury to the thyroid [10]. In our case, TSH was increased and free T4 was normal. The patient had increased lethargy and upper arm weakness, and testing for antibodies was negative. These findings led to a diagnosis of CPI-induced primary hypothyroidism secondary to thyroid injury with an intact central response.

After initial treatment cessation with a two-month washout period, ipilimumab and nivolumab combination therapy was started. The patient then presented with DKA and significantly decreased C-peptide, leading to the diagnosis of insulin-deficient DM. The incidence of DM1 related to CPI in patients on CPI is 0.2% to 1.1% [11]. In our case, the C-peptide level was significantly decreased, and glucagon level was normal, suggesting that damaged beta cells, along with functional alpha cells, led to DM1. This process had been attributed to increased T-cell autoreactivity causing beta-cell destruction [9]. But the exact mechanism is unknown. One study showed a correlation between increased B-cell activity and irAEs, suggesting the role of B-cells in autoimmune DM [10]. It seems that CPI-induced DM1 and other irAEs are caused by both T- and B-cell activity. Further studies are needed to draw definitive conclusions.

Given the temporal dynamics and doses of CPIs in this case, either or both CPI combination therapy trials may have led to DM1. During treatment with durvalumab and tremelimumab (first combination), we found slightly elevated lipase (1.5-2x upper limit of normal) over the course of therapy. Although it never increased enough to elicit suspicion for pancreatitis, this could represent a sign of mild pancreatic injury, and later ipilimumab and nivolumab, second combination therapy, served as a final trigger for overt DM1 presenting as DKA [11]. Other evidence in favor of a gradual onset of DM1 is that the HbA1C was elevated to 8.5%, which estimates average blood glucose of 180 mg/dl over the preceding three-month span. Hence, it is possible that glucose levels started rising before the second combination therapy, although it is also possible that elevated HbA1C may have been due to hyperglycemia over the short term than mild hyperglycemia over months [12]. This finding further suggests that there was added damage by ipilimumab and nivolumab on the pancreas on top of mild chronic pancreatic injury caused by durvalumab and tremelimumab.

## Conclusions

With the increasing number of CPI options, more and more patients will receive multiple trials of different CPIs. Hence, this possibility of irAEs from one trial decreasing the threshold for severe irAE from consecutive trials is very concerning. Clinicians should have a low threshold for diagnosis of immune-related pathologies (insulin-dependent DM in our case) in patients with a CPI. We need to be careful about the sequential use of combination CPIs to set treatment goals with the patient’s goals of optimal care.
